# Correction: Antibody recruiting molecules (ARMs): synthetic immunotherapeutics to fight cancer

**DOI:** 10.1039/d1cb90021h

**Published:** 2021-07-22

**Authors:** Silvia Achilli, Nathalie Berthet, Olivier Renaudet

**Affiliations:** Univ. Grenoble Alpes, CNRS, DCM UMR 5250 F-38000 Grenoble France nathalie.berthet@univ-grenoble-alpes.fr olivier.renaudet@univ-grenoble-alpes.fr

## Abstract

Correction for ‘Antibody recruiting molecules (ARMs): synthetic immunotherapeutics to fight cancer’ by Silvia Achilli *et al.*, *RSC Chem. Biol.*, 2021, **2**, 713–724. DOI: 10.1039/d1cb00007a.

The authors regret mistakes in the structures depicted in Table 1, entry A and Table 2, entry B. The correct version of both tables are shown below. The conclusions of the paper have not been affected.

**Table 1:** Examples of ARMs against cancer cells

Entry	Tumor target	ARM valency	ABM	TBM	ARM structure
A^41^	uPAR	Mono	DNP	uPAR inhibitor	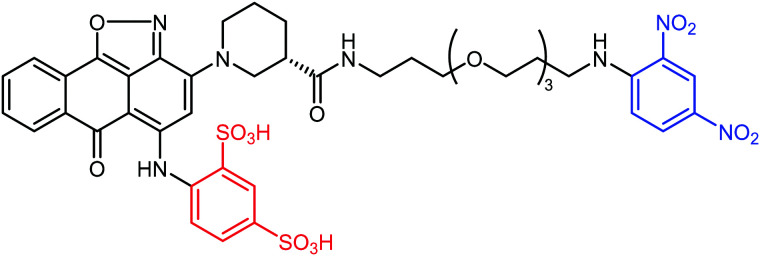
B^46^	VEGF/osteopotin	Mono	DNP	anti-VEGF and anti-osteopontin aptamer	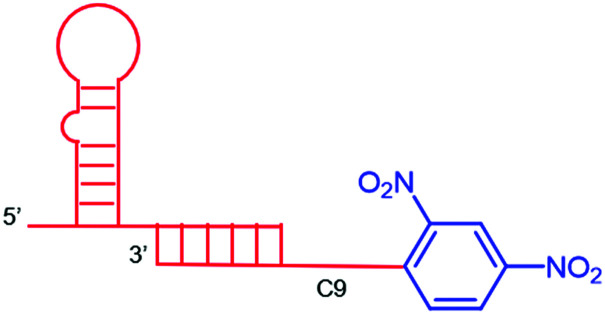
C^51^	Folate receptor	Mono	Fc-binding cyclic peptide	Folic acid	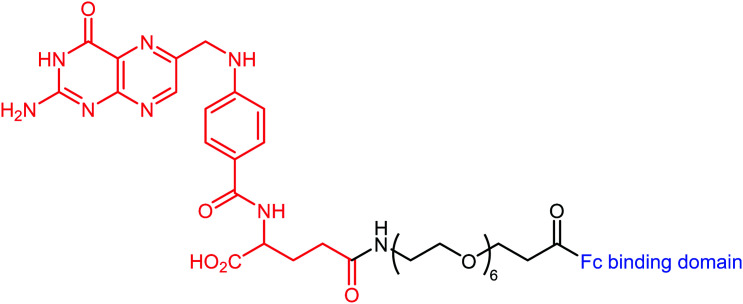
D^55^	PSMA	Mono	DNP	Glutamate urea	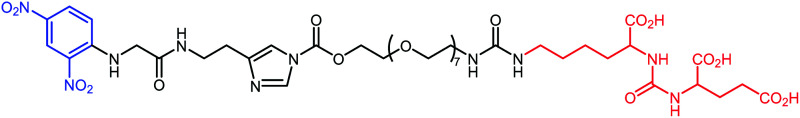
E^56^	α_v_β_3_ integrins	Multi	l-Rha	cRGD	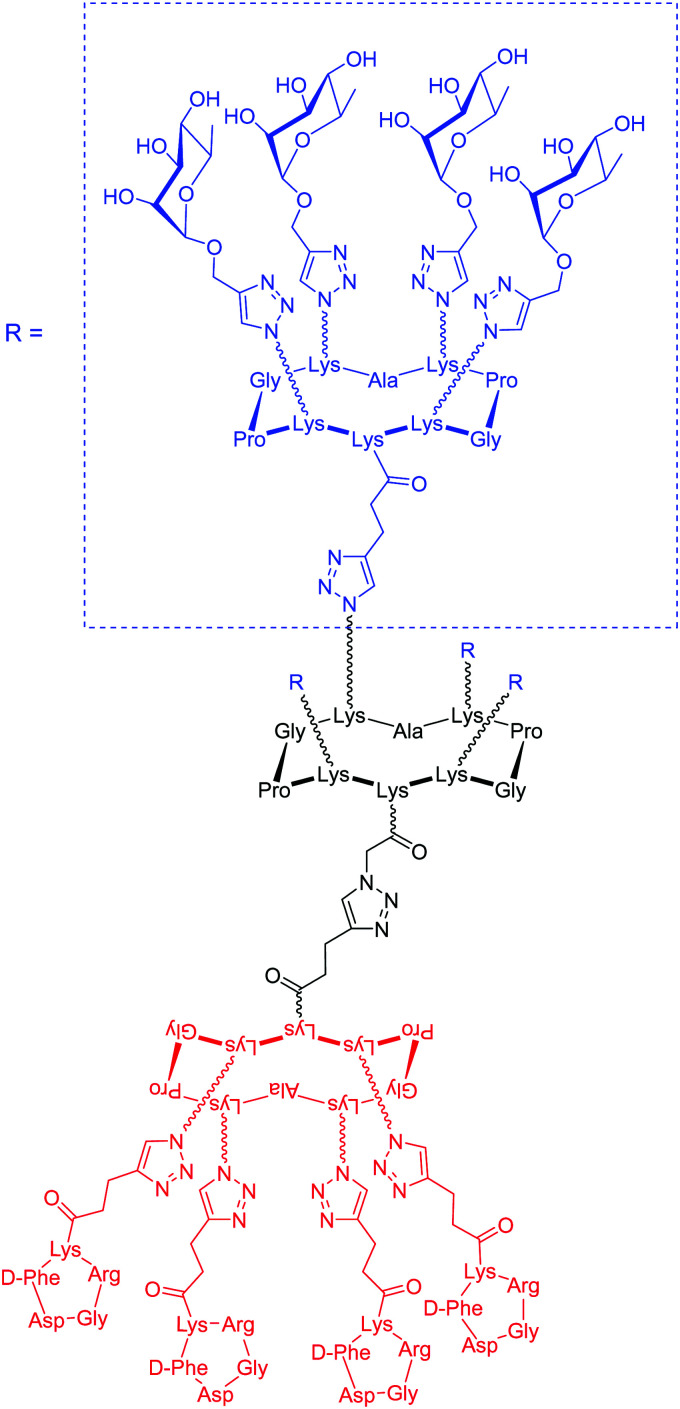

**Table 2:** Example of ARMs using unspecific targeting of the cancer cell membrane with lipid anchor

Entry	Tumor target	ARM valency	ABM	TBM	ARM structure
A^12^	uPAR	Multi	l-Rha	CholA anchor	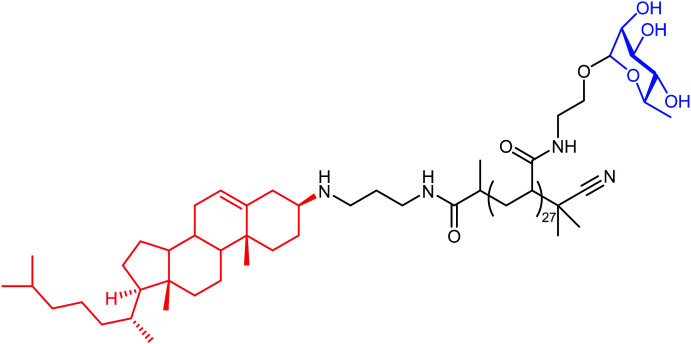
B^65^	VEGF/osteopotin	Multi	DNP	Di-alkyl anchor	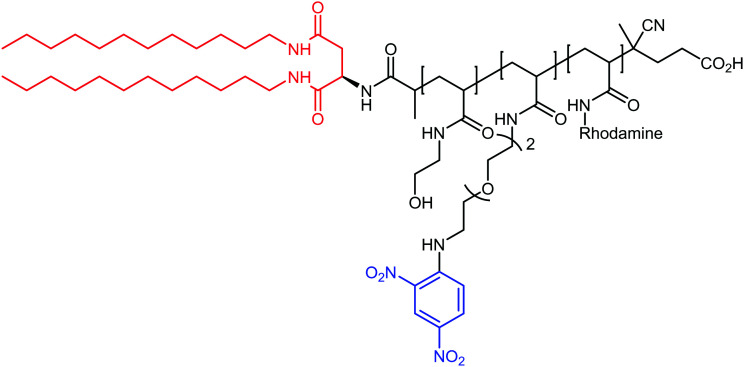

The Royal Society of Chemistry apologises for these errors and any consequent inconvenience to authors and readers.

## Supplementary Material

